# THE NEED FOR PRAGMATIC PROGRAMMING FOR CAREGIVERS: EVIDENCE FROM MANAGING MONEY: A CAREGIVER’S GUIDE TO FINANCES

**DOI:** 10.1093/geroni/igae098.1900

**Published:** 2024-12-31

**Authors:** Claire Grant, Katherine Judge, Lauren Stratton, Monica Moreno, Sam Fazio

**Affiliations:** Cleveland State University, Cleveland, Ohio, United States; Cleveland State University, Cleveland, Ohio, United States; Alzheimer’s Association, Chicago, Illinois, United States; Alzheimer’s Association, Chicago, Illinois, United States; Alzheimer’s Association, Chicago, Illinois, United States

## Abstract

Several key issues remain in the caregiving intervention literature. Programs must seamlessly transition and scale into existing settings with plans for long-term implementation. Programs should be accessible, digestible, and efficient for caregivers while still delivering meaningful results. To address these issues, conceptualization and development should be guided by end-use scenarios. Managing Money: A Caregiver’s Guide to Finances was developed as an online program for caregivers to address the need for pragmatic and scalable programs focused on the financial impacts of caregiving. Synchronous (community educator-led) and asynchronous (self-paced) versions of the one-hour online program were offered. Topics include conversations about finances, assessing needs, and finding financial support. Printable tools were also provided. The synchronous program was evaluated as an RCT (N=179). The mean age was 55.03 (SD=15.34), 73.2% White; 48.6% cared for a parent/grandparent; and 36.3% cared for a spouse. Long-term effects were found in reducing unmet needs and distress around finances and increasing the likelihood to act to improve financial situations. The asynchronous version was evaluated in a one-arm study with 146 caregivers. The mean age was 45.85 (SD=5.65); 75% were white, and 91% cared for a parent/grandparent. Both versions were found to be acceptable and feasible. On a Likert scale ranging from 1 (strongly disagree) to 5 (strongly agree) participants reported the program identified financial challenges (M=4.09, SD=.75/M=3.81, SD=.79) and would recommend the program (M=4.50, SD=.86/M=3.91, SD=.84). Discussion will outline features of program development, rollout, and scalability. Ongoing need and recommendations for pragmatic programming will also be discussed.

